# Multiscale Complexity and Irreversibility of Non-Stationary Time Series in Commodity Futures Markets

**DOI:** 10.3390/e28090984

**Published:** 2026-09-03

**Authors:** Xia Zhao, Kaicheng Xie

**Affiliations:** Institute of Food and Strategic Reserves, Nanjing University of Finance and Economics, Nanjing 210023, China

**Keywords:** futures markets, multifractal analysis, time irreversibility, multiscale entropy, structural breaks, geopolitical risk

## Abstract

Commodity futures markets exhibit pronounced non-stationarity, nonlinearity, and multifractal characteristics that challenge traditional linear models. We employ a multiscale framework integrating four methodologies—MF-DCCA, PG irreversibility index, MSWPE, and JS-divergence segmentation—to analyze these features using daily closing prices for WTI crude oil, agricultural commodities (US soybeans, meal, oil, and wheat), the US dollar index, and Chinese No. 2 soybeans, spanning from 2 January 2018 to 1 October 2025, sourced from Investing.com and Matteo Iacoviello’s GPR database. The analysis yields three key results. First, scale dependence varies across commodities and is shaped by supply adjustment elasticity: energy markets show scale-dependent amplification and directional sign reversals, while agricultural markets maintain near-monofractal structures. Second, multiple methods converge on a characteristic time scale of approximately 20 days, linking physical logistics rhythms with financial pricing. Third, the persistence of structural reconstruction after shocks depends on systemic penetration depth, with exogenous macroeconomic uncertainty exerting stronger and more lasting effects than market-internal events. Together, supply elasticity, physical logistics rhythms, and systemic penetration depth constitute the three fundamental determinants of nonlinear commodity futures dynamics, with implications for cross-commodity allocation, multi-horizon risk management, and geopolitical scenario analysis.

## 1. Introduction

Commodity futures markets exemplify complex systems, yet the ways in which their price dynamics depend on observation scale have not been systematically characterized. Prices in these markets reflect a confluence of factors: supply–demand fundamentals, macroeconomic conditions, geopolitical risks, inventory fluctuations, and market microstructure. These forces operate across markedly different time horizons—intraday swings driven by algorithmic trading, seasonal agricultural cycles and OPEC adjustments spanning months, and multi-year shifts in infrastructure investment and global trade architecture. The variation across scales, combined with trend persistence and structural heterogeneity across commodities, tends to obscure the actual relationships and causal pathways among variables. Complex systems, as Waldrop observed, are defined less by their components than by the interactions among them and the emergent properties that follow—properties not inferable from isolated observations [1]. Commodity futures markets fit this description. A diverse set of participants—producers, consumers, speculators, arbitrageurs, hedgers—operate under different information sets, risk preferences, and time horizons. The resulting price dynamics are distinctly nonlinear: long-range correlations, volatility clustering, intermittent bursts, and asymmetric responses to information shocks all appear. These markets are fundamentally non-equilibrium systems. Prices are repeatedly pushed away from equilibrium by external shocks and internal feedback, then gradually return through intricate, multi-stage adjustment processes. How these adjustments vary with the chosen observation scale and with the structural attributes of different commodities is the central question this study addresses.

Two broad frameworks underlie much of the analysis in this area: fractal theory and information theory. Mandelbrot’s fractal theory [2] suggests that many natural and social phenomena exhibit self-similarity under scale changes, captured by the Hurst exponent *H*, with values above 0.5 indicating persistence and below 0.5 anti-persistence. A single exponent, however, seldom suffices for real-world data; financial signals typically show different scaling behavior across small versus large fluctuations, a property known as multifractality. The multifractal spectrum, summarized by the generalized Hurst exponent h(q), captures the range of local scaling exponents present in the data [3,4,5]. The multifractal properties of commodity markets have received growing attention. Ftiti et al. [6] examined oil and gas futures market efficiency through the lens of multifractality. Mensi et al. [7] extended this line of inquiry to metals futures during financial crises. Lahmiri [8] combined multifractal- and entropy-based approaches to study energy markets under COVID-19. One empirical pattern we explore below is that multifractality itself shifts with commodity type and with the time scale examined.

The introduction of detrended fluctuation analysis (DFA) marked a significant advance by removing non-stationary trends that distort correlation estimates [9,10]. Kantelhardt and colleagues generalized this to multifractal DFA (MFDFA) [11], enabling multifractal analysis of non-stationary series. Subsequently, Podobnik and Stanley [12] introduced detrended cross-correlation analysis (DCCA), and Zhou [13] extended it to MF-DCCA, allowing scale-dependent cross-multifractal analysis between signal pairs. Recent methodological advances have improved robustness on short or noisy records [14] and clarified conditions for reliable estimation [15]. Extensions to moving-average and time-delayed formulations have also been developed [16,17,18]. Yet fractal methods, while powerful for correlation structure and scale invariance, are less suited to capturing ordinal patterns, informational hierarchies, or directional asymmetries. Information-theoretic tools fill some of these gaps.

Commodity markets also exhibit rich nonlinear dependence structures beyond multifractality. Chen et al. [19] used dynamic copula models to capture time-varying tail dependencies across commodity futures. Dahal and Mattos [20] examined nonlinear deterministic dynamics in commodity prices. Ren et al. [21] showed that economic policy uncertainty drives nonlinear risk spillovers across commodity sectors, while Hanif et al. [22] documented asymmetric tail dependencies in portfolios combining metals and agricultural futures. Together, these studies suggest that linear models and Gaussian assumptions are inadequate for understanding commodity market linkages.

Costa and co-authors [23,24] observed that conventional entropy measures assign their highest values to white noise and their lowest to perfectly ordered sequences, motivating the development of multiscale entropy (MSE), which computes sample entropy at progressively coarse-grained scales [25]. Refinements such as composite MSE (CMSE) and refined composite MSE (RCMSE) addressed biases in short records. Zhou et al. [26] and Fu, Sun, and Liu [27] applied MSE-based tools to financial and carbon markets, while Zhao et al. [28] and Xue et al. [29] employed transfer entropy networks to capture risk propagation and cross-market interconnectedness. A related development is permutation entropy, due to Bandt and Pompe [30], which relies on the relative ordering of successive observations rather than actual magnitudes, making it robust against noise and computationally light [30]. Zhao, Shang, and Wang [31] adapted this framework to quantify information interactions on ordinal patterns of stock returns. Combining permutation entropy with multiscale coarse-graining gives multiscale permutation entropy (MSPE), which tracks how ordinal structure changes across scales. Orlando [32] found that combining entropy-based measures with multifractal analysis characterizes financial market complexity more comprehensively than either approach alone. A complication is trend interference: trends can distort correlation estimates and amplify noise in entropy computations, especially when series contain pronounced deterministic movements.

Several preprocessing approaches, including empirical mode decomposition (EMD) and Fourier-based filtering, have been proposed to address trend interference in entropy analysis [33,34,35]. We adopt the standard coarse-graining procedure of Costa et al. [23], as our focus is on the scale-resolved complexity of the original series rather than detrended residuals. In a related development, Stylianou et al. [36] introduced a multiscale detrended cross-correlation coefficient that provides a standardized measure of coupling across scales, complementing our analytical approach. Miranda, Dolfin, Kapetanios, and Leonida [37] introduced a multiscale measure of network reconfiguration based on DCCA and minimum spanning tree filtering. The measure captures how cross-correlation network topology changes across investment horizons and distinguishes between stressed and stable market regimes.

Time series irreversibility introduces a further dimension. The question is whether reversing a time series leaves its statistical properties unchanged. If not, the series exhibits directional asymmetry. Irreversibility typically signals non-equilibrium dynamics—systems driven externally or subject to internal dissipation. Porat and Friedland showed that irreversibility can be captured through asymmetry in the distribution of increments, offering a way to detect directional information flow and non-conservative forces. In financial settings, irreversibility has been linked to leverage effects, volatility asymmetries, and the uneven impact of positive versus negative news—all features that symmetric equilibrium models cannot reproduce. Zhao, Shang, and Wang [38] developed a framework to measure the asymmetric contributions of subsystems, finding that uneven contribution patterns reveal something about the system’s internal organization. The degree of irreversibility is not static; it shifts with market conditions, the chosen investment horizon, and the type of shocks involved. Major exogenous shocks, in particular, tend to leave structural breaks that push the system into new, non-stationary regimes. Zanin and Papo [39] recently surveyed methods for assessing multiscale irreversibility, while Chen et al. [19] proposed a trend-pattern-length approach that complements amplitude-based measures. The key question for our purposes is whether directional asymmetry depends on scale—and if so, whether a characteristic time scale exists.

Structural break detection adds yet another layer. Abrupt shifts in the data-generating process often point to regime changes driven by exogenous shocks or internal transitions. One segmentation method, based on Jensen–Shannon (JS) divergence, offers a non-parametric way to locate change points by maximizing distributional divergence across adjacent windows. Wang, Shang, Lin, and Chen [40] introduced segmented inner composition alignment to detect coupling patterns in complex systems, even when these patterns vary over time. For commodity markets, this method is particularly relevant: geopolitical events—wars, sanctions, supply disruptions, trade disputes—can trigger sudden and long-lasting changes in price behavior and cross-market linkages. Mapping detected breaks to external events builds an empirical bridge between geopolitical risk and market dynamics; this mapping is validated later by aligning breakpoints with the GPR index.

Recent work has highlighted the role of geopolitical risk in commodity markets. Zheng et al. [41] documented that geopolitical risk significantly affects the volatility of natural resource commodity futures in China. Wang et al. [42] showed that the Russia–Ukraine war amplified systemic risk across commodity markets through interconnected channels. Cheng, Liao, and Pan [43] estimated a geopolitical risk premium in commodity futures, finding that threats and realized acts have distinct pricing implications. Yang, Yang, and Feng [44] quantified the resilience of commodity futures markets to geopolitical shocks, offering a framework for assessing market vulnerability across commodities. Together, these studies indicate that geopolitical risk is a structural driver of commodity market dynamics, not merely transient noise.

A central challenge in this literature is the economic interpretation of complexity measures. Multifractality and entropy-based statistics are often reported as purely statistical descriptors without clear economic meaning. Recent research has begun to bridge this gap. Fernandes et al. [45] linked complexity measures to boom-and-bust dynamics by assessing disorder and similarity in commodity prices over economic cycles. Yang, Feng, and Yang [46] examined multiscale dynamic interdependency between China’s crude oil futures and petrochemical-related commodity futures, offering an industry-chain perspective on nonlinear linkages. These studies indicate that complexity measures, when interpreted within an economic framework, can reveal fundamental supply-chain structures, participant heterogeneity, and shock transmission mechanisms. We ground our complexity measures in three economic constructs—supply elasticity, logistics rhythms, and systemic penetration depth—to move beyond purely statistical description.

While each of these four methodological streams has been applied individually to financial data, their integration within a single analytical framework—and, more importantly, whether they converge on a common set of empirical regularities—has received little attention. We bring these threads—multifractal cross-correlation analysis, entropy-based complexity measures, irreversibility diagnostics, and structural break detection—together in a single framework. The aim is to move beyond piecewise characterizations and toward a more integrated view of how commodity futures markets behave as multi-scale systems. Three questions structure the empirical work: (i) How does cross-market coupling change across time scales, and what explains the differences across commodities? (ii) Does directional asymmetry shift systematically with the investment horizon, and if so, at what characteristic time scale? (iii) Do geopolitical shocks induce persistent structural changes beyond short-lived volatility spikes, and does their persistence depend on the depth of systemic penetration—that is, the extent to which a shock propagates beyond the market in which it originates?

To this end, we use MF-DCCA to measure scale-dependent cross-correlations between WTI crude oil, the US dollar index, and US–China soybean prices to examine whether correlation strength and multifractal breadth vary with horizon and whether supply-side attributes (concentration versus substitutability) drive these variations. We apply the PG irreversibility index across multiple scales to test whether sign reversal occurs, which would signal a shift in the dominant direction of price adjustment. We detect structural breaks in WTI prices via JS divergence and align them with GPR-indexed events to test the connection between geopolitical risk and structural market change. We also apply MSWPE to the soybean crushing chain to test whether downstream products show lower complexity than upstream raw materials—a pattern that would clarify how shocks propagate along production chains and offer indirect evidence on systemic penetration depth.

We build the analytical framework around a common hypothesis: supply adjustment elasticity, physical logistics rhythms, and systemic penetration depth jointly govern the nonlinear dynamics of commodity futures markets. Four methods test distinct facets of this hypothesis. Each method is applied to the commodity subset that most clearly reveals the mechanism of interest; WTI crude oil serves as the common anchor across all methods. The empirical analysis is guided by four specific hypotheses:

**H1 (primary).**  *The MF-DCCA spectrum width for WTI–DXY peaks at the 20-day scale.*

**H2 (primary).**  *The PG irreversibility index for WTI undergoes a sign reversal between the 10-day and 20-day scales.*

**H3 (secondary).**  *The MSWPE entropy of soybean oil drops at the 40-day scale relative to soybeans and soybean meal.*

**H4 (secondary).**  *JS divergence breakpoints align with major geopolitical events at a rate exceeding chance.*

All other scale-specific comparisons and cross-commodity contrasts are treated as exploratory. For the primary hypotheses, we report bootstrap confidence intervals and *p*-values. For secondary hypotheses, we apply multiple-testing corrections where applicable and interpret the results with appropriate caution.

The rest of this paper is structured as follows. Section 2 lays out the methodological framework and describes the data and preprocessing steps. Section 3 presents the empirical findings. Section 4 discusses their implications and limitations. Section 5 concludes.

## 2. Data and Methods

### 2.1. Data and Preprocessing

The daily data cover the period from 2 January 2018 to 1 October 2025, with a total of 2022 trading days. Table 1 lists the eight variables used in our empirical analysis, grouped into three categories: macro-financial indicators, commodity futures prices, and the geopolitical risk index employed for event interpretation. The GPR Index, constructed by Caldara and Iacoviello [47], measures geopolitical tensions by counting the number of newspaper articles covering adverse geopolitical events. This index is commonly used in empirical finance to capture exogenous political shocks to commodity markets.

All futures data are daily closing prices for near-month contracts from Investing.com. The contracts are WTI crude oil (NYMEX: CL), US soybeans (CBOT: ZS), US soybean meal (CBOT: ZM), US soybean oil (CBOT: ZL), US wheat (CBOT: ZW), and DCE No. 2 Soybean (ticker: B). To build continuous price series, we use a 5-day roll window that transitions from the expiring contract to the next-month contract during the five trading days before the first notice date. We apply ratio-adjustment (proportional back-adjustment): historical prices are multiplied by the ratio of the new contract price to the old contract price at the roll date. This preserves percentage changes and prevents artificial level shifts. We exclude observations on expiration days to avoid microstructure distortions.

All analyses use the raw price series $P_{t}$ as inputs. Each method internally applies the transformation appropriate to its mathematical requirements: MF-DCCA constructs integrated profiles from raw prices; PG irreversibility and MSWPE operate on the raw series directly; and JS divergence detects structural breaks using raw price levels, with an internal log-transform for histogram estimation. We confirm that the WTI price series used in this study is the near-month futures contract (CL1) obtained from Investing.com, and that the negative price event of 20 April 2020 is not present in our data, as the continuous futures series employs a ratio-adjusted roll procedure. All prices in our sample are strictly positive, and all transformations are therefore well-defined.

For the US–China soybean cross-market analysis, we use only days when both CBOT and DCE were open, avoiding artificial zero returns from forward-filling during non-overlapping holidays. We align Chinese soybean prices with the previous day’s US prices to respect the actual sequence of information flow. We keep Chinese No. 2 soybean prices in RMB (CNY) and US soybean prices in USD. We do not convert the Chinese prices into USD because the exchange rate itself is a channel of cross-market arbitrage friction, and we already capture it separately through the Dollar_Index variable.

Missing values are forward-filled, yielding a final sample of 2022 daily observations. Augmented Dickey–Fuller (ADF) tests show that the raw price series are integrated of order one, which is consistent with the non-stationary nature of commodity futures prices. The methodologies used in this study do not require stationarity, as they are designed for such data.

The four methods are applied to the same raw price series $P_{t}$, with each transformation determined by the method’s mathematical structure. Table 2 lists the input series, transformation, statistic, and economic hypothesis for each approach. They are chosen for their complementarity and their ability to handle non-stationary financial data—a property that distinguishes them from common alternatives. Wavelet coherence, for instance, requires stationarity in the cross-spectrum and is rarely reliable during extreme episodes; transfer entropy depends on binning choices and does not reveal scale-dependent patterns; recurrence quantification analysis and dynamic time warping provide no information about multifractal properties; and network-based methods rely on coarse temporal aggregations that obscure characteristic frequencies. In contrast, the selected methods address distinct dimensions of the problem: MF-DCCA quantifies scale-dependent cross-correlations, MSWPE measures univariate complexity, PG captures directional asymmetry, and JS divergence detects regime shifts. Used together, they offer a broader perspective than any single method could provide.

### 2.2. Unified Analytical Framework and Common Hypothesis

Before detailing the individual methodologies, we clarify the common hypothesis that ties them together and drives the commodity-specific design choices. We hypothesize that the nonlinear dynamics of commodity futures markets are jointly shaped by three fundamental determinants.

First, supply adjustment elasticity governs the degree of scale-dependent heterogeneity in cross-market linkages. Low elasticity commodities—crude oil, for instance—should display stronger scale-dependent amplification of cross-correlations, while high elasticity commodities—globally tradable soybeans—should preserve near-monofractal structures across scales.

Second, physical logistics rhythms—shipping times, inventory turnover, and production cycles—introduce characteristic time scales into price dynamics, appearing as directional sign reversals that mark the frequency at which markets digest shocks and complete directional turns.

Third, systemic penetration depth determines how long structural reconstruction persists after a shock. The persistence depends on whether the shock propagates beyond its market of origin through demand expectations, policy channels, and risk premia. Shocks that penetrate more deeply should induce more persistent shifts in the price-generating process.

We test this tripartite hypothesis with four complementary methodologies. Each method is applied to the commodity subset that best isolates the mechanism under investigation; WTI crude oil serves as the common anchor across all four methods, enabling cross-method comparison. Table 3 maps each method to the hypothesis facet it tests, the commodity series it uses, and the economic mechanism it interrogates.

### 2.3. Multifractal Detrended Cross-Correlation Analysis (MF-DCCA)

We employ MF-DCCA, an extension of detrended fluctuation analysis (DFA), to capture scale-dependent cross-correlations between energy and agricultural markets and to quantify the multifractal properties of cross-correlations between two non-stationary series [13]. Unlike conventional correlation measures that assume linearity and stationarity, MF-DCCA accommodates non-stationary trends and distinguishes between cross-correlations of small versus large fluctuations via the generalized Hurst exponent $h_{xy}\left(q\right)$. This enables us to test whether cross-market linkages differ between turbulent periods (captured by q>0) and relatively quiet periods (q<0).

The computational procedure is as follows. Given two series $\left\{x_{i}\right\}$ and $\left\{y_{i}\right\}$, each of length *N*, we first construct their integrated profiles:

$$X\left(i\right)=\sum_{k=1}^{i}\left(x_{k}-\bar{x}\right),\;Y\left(i\right)=\sum_{k=1}^{i}\left(y_{k}-\bar{y}\right),\;i=1,2,\ldots ,N$$

Each profile is divided into $N_{s}=\left\lfloor N/s\right\rfloor$ non-overlapping segments of length *s*. For each segment *v*, a polynomial of order *m* is fitted to the local trend and subtracted. For s=2, we use first-order detrending (m=1); for all larger scales, we use quadratic detrending (m=2). This hybrid choice keeps the detrending order compatible with the segment length across scales. The detrended covariance is computed as follows:

$$F^{2}\left(s,v\right)=\frac{1}{s}\sum_{j=1}^{s}\left[X_{v}\left(j\right)-{\tilde{X}}_{v}\left(j\right)\right]\left[Y_{v}\left(j\right)-{\tilde{Y}}_{v}\left(j\right)\right]$$

where ${\tilde{X}}_{v}\left(j\right)$ and ${\tilde{Y}}_{v}\left(j\right)$ are the fitted polynomial trends. The *q*-th order fluctuation function is then

$$F_{q}\left(s\right)={\left[\frac{1}{2N_{s}}\sum_{v=1}^{2N_{s}}{\left(F^{2}\left(s,v\right)\right)}^{q/2}\right]}^{1/q},\;q\neq 0$$

For negative values of the detrended covariance $F^{2}\left(s,v\right)$, we adopt the sign-preserving convention following standard practice in the multifractal literature:

$${\left(F^{2}\left(s,v\right)\right)}^{q/2}=\left\{\begin{array}{cc} {\left(F^{2}\left(s,v\right)\right)}^{q/2}, & F^{2}\left(s,v\right)\geq 0,\\ -|F^{2}{\left(s,v\right)|}^{q/2}, & F^{2}\left(s,v\right)<0\;\mathrm{and}\;q\;\mathrm{is}\;\mathrm{odd},\\ |F^{2}{\left(s,v\right)|}^{q/2}, & F^{2}\left(s,v\right)<0\;\mathrm{and}\;q\;\mathrm{is}\;\mathrm{even}. \end{array}\right.$$

For q=0, we treat the expression via the limiting case $F_{0}\left(s\right)=\exp\left(\frac{1}{4N_{s}}\sum_{v=1}^{2N_{s}}\ln\left(F^{2}\left(s,v\right)\right)\right)$.

We estimate local scaling exponents to obtain scale-resolved multifractal characteristics, rather than fitting a single global exponent across all scales. For each target scale $s_{0}\in \left\{2,5,10,20,40,60\right\}$, we regress $\log F_{q}\left(s\right)$ against logs over a local window $\left[s_{0}/2,\;2s_{0}\right]$ (i.e., $\log s\in [\log\left(s_{0}/2\right),\;\log\left(2s_{0}\right)]$ in log-space). The slope coefficient is taken as the local scaling exponent $h_{xy}^{\left(\mathrm{local}\right)}\left(q;s_{0}\right)$. The local window contains 4 to 6 scale points, depending on the target scale, and the adjusted $R^{2}$ values are consistently above 0.95. This procedure follows Kantelhardt et al. [11], who used a similar local regression approach for scale-resolved multifractal analysis.

If $F_{q}\left(s\right)$ and *s* satisfy the power-law relation $F_{q}\left(s\right)∼s^{h_{xy}\left(q\right)}$, then $h_{xy}\left(q\right)$ is the generalized cross-Hurst exponent. When q=2, this reduces to the standard Hurst exponent *H*: 0.5 indicates uncorrelated behavior, values above 0.5 indicate persistence, and values below 0.5 indicate anti-persistence. The multifractal spectrum width $\Delta h=h_{xy}\left(q_{\min}\right)-h_{xy}\left(q_{\max}\right)$ measures the degree of multifractality—larger values indicate richer multifractal structure. The singularity spectrum f(α) is obtained via the Legendre transform:

$$\alpha =h\left(q\right)+qh^{\prime }\left(q\right),\;f\left(\alpha \right)=q\left[\alpha -h\left(q\right)\right]+1$$

We compute MF-DCCA over six scales: s∈{2,5,10,20,40,60} days, covering intra-week, monthly, and quarterly investment horizons. The fluctuation order is set to q∈[−5,−4,…,4,5], a range commonly adopted in the multifractal literature [11,13,18]. This range captures the asymptotic scaling behavior without entering the unstable region associated with extreme *q* values.

### 2.4. Porat–Friedland (PG) Irreversibility Index

Time irreversibility refers to whether the statistical properties of a time series remain invariant under time reversal. When they do not, the system is out of equilibrium, typically indicating dissipative dynamics [48]. In financial markets, time irreversibility manifests as asymmetric price responses to upward versus downward movements. This asymmetry is commonly attributed to the leverage effect—the negative correlation between volatility and returns—and to the differential impact of positive versus negative news on volatility.

We use the PG index to quantify this asymmetry because it is structurally simple, interpretable, and captures both directional bias and magnitude asymmetry in a single scalar.

Given a sequence $\left\{z_{t}\right\}$, define the difference $\Delta_{t}=z_{t}-z_{t-1}$. Let $p_{+}$ and $p_{-}$ denote the proportions of positive and negative differences, and $\mu_{+}$ and $\mu_{-}$ their respective means. The PG index is defined as follows:

$$PG=\frac{1}{2}\left[p_{+}\log\left(\frac{p_{+}}{p_{-}}\right)+\frac{\mu_{+}-\mu_{-}}{\mu_{+}+\mu_{-}}\right]$$

The first term captures directional bias; the second captures magnitude asymmetry. Positive PG values indicate that upward movements are faster or more frequent than downward movements; negative values indicate the opposite.

**Component decomposition.** The PG index decomposes into two additive components: a frequency component $PG_{\mathrm{freq}}=\frac{1}{2}p_{+}\log\left(p_{+}/p_{-}\right)$ and a magnitude component $PG_{\mathrm{mag}}=\frac{1}{2}\left(\mu_{+}-\mu_{-}\right)/\left(\mu_{+}+\mu_{-}\right)$. The decomposition shows whether irreversibility comes from directional frequency or magnitude asymmetry. A positive PG can arise from more frequent upward moves, larger average upward moves, or both. We report both components alongside the aggregate PG index for economic interpretation.

To examine whether irreversibility varies with scale, we compute the PG index separately at each scale s∈{2,5,10,20,40,60}. The coarse-graining procedure follows the same logic as in multiscale entropy analysis [23]:

$$z_{j}^{\left(s\right)}=\frac{1}{s}\sum_{i=(j-1)s+1}^{js}z_{i}$$

where $z^{\left(s\right)}$ is the coarse-grained series at scale *s*. We examine whether the direction of irreversibility—and hence the dominant forces driving price dynamics—changes with the observation horizon, a question that has not been systematically examined in commodity markets.

**Statistical inference.** We assess the sampling uncertainty of the PG estimates using a moving-block bootstrap (block length =100 days, N=1000 resamples) that preserves serial dependence and conditional heteroskedasticity. For each scale *s*, we construct 95% bootstrap confidence intervals for $PG_{s}$ and test $H_{0}:PG_{s}=0$ against $H_{1}:PG_{s}\neq 0$. We also test whether adjacent scales differ significantly using a bootstrap difference test for $H_{0}:PG_{s}=PG_{s^{\prime }}$. These procedures provide a statistical basis for the directional interpretations.

### 2.5. Multiscale Weighted Permutation Entropy (MSWPE)

Permutation entropy measures complexity by mapping consecutive values to ordinal patterns. The method is robust, computationally efficient, and insensitive to amplitude noise [30]. Weighted permutation entropy extends the standard version by incorporating amplitude information through a variance-based weighting mechanism. This reduces sensitivity to observational noise and enhances robustness, making it particularly suitable for futures return series, which are known to exhibit heavy-tailed features [49].

For an embedding dimension *d* and delay τ=1, each vector $\left[z_{t},z_{t+\tau },\ldots ,z_{t+(d-1)\tau }\right]$ is mapped to a permutation π of order *d* based on the rank order of its elements. For each pattern $\pi_{i}$, the weighted relative frequency is as follows:

$$p\left(\pi_{i}\right)=\frac{\sum_{t}w_{t}\cdot 1_{pattern_{t}=\pi_{i}}}{\sum_{t}w_{t}}$$

where the weight $w_{t}$ is the variance of the embedded vector:

$$w_{t}=\frac{1}{d}\sum_{j=1}^{d}{\left(z_{t+(j-1)\tau }-{\bar{z}}_{t}\right)}^{2},$$

with ${\bar{z}}_{t}$ denoting the mean of the embedded vector. The weighted permutation entropy is then

$$H_{w}\left(d\right)=-\sum_{i=1}^{d!}p\left(\pi_{i}\right)\log_{2}p\left(\pi_{i}\right)$$

We set d=3, which yields d!=6 possible ordinal patterns. This choice is motivated by two considerations. First, the sample size N=2022 is far larger than d!, satisfying the statistical requirement N≫d! [30]. Second, this value provides a reasonable balance between pattern richness and estimation reliability. Higher entropy values indicate greater irregularity and lower predictability.

**Effective sample size and finite-sample robustness.** After coarse-graining at scale *s*, the effective sample size is reduced to ⌊N/s⌋. For s=40 and s=60, this yields approximately 50 and 33 coarse-grained observations, respectively. Estimating the six ordinal pattern probabilities (d!=6) from such sample sizes may introduce finite-sample bias. We use two complementary checks:

1. **Composite coarse-graining**: Following the composite multiscale entropy approach [50], we generate multiple coarse-grained series for each scale by shifting the starting point of the coarse-graining procedure, and average the entropy estimates across these realizations. This reduces estimation variance and addresses finite-sample bias.
2. **Alternative embedding dimensions**: We compute MSWPE with d=2 and d=4 in addition to the baseline d=3 to assess whether the qualitative pattern—particularly the 40-day entropy drop for soybean oil—is robust to the choice of embedding dimension.

We compute MSWPE across the same set of scales s∈{2,5,10,20,40,60}, ensuring consistency with the MF-DCCA and PG analyses.

### 2.6. Jensen–Shannon Divergence for Structural Break Detection

The Jensen–Shannon divergence measures the similarity between two probability distributions *P* and *Q* [51]:

$$JS\left(P||Q\right)=\frac{1}{2}D_{KL}\left(P||M\right)+\frac{1}{2}D_{KL}\left(Q||M\right)$$

where $M=\frac{1}{2}\left(P+Q\right)$ and $D_{KL}$ is the Kullback–Leibler divergence. JS∈[0,ln2], with JS=0 indicating identical distributions.

For structural break detection, we employ a sliding window of width w=120 trading days—approximately six months. This window length balances two requirements: sufficient observations for reliable distribution estimation and reasonable timeliness in break detection. For each position *t*, we compare the empirical distributions of the left segment [t−w,t] and the right segment [t,t+w]. The raw price series $P_{t}$ is used to detect level shifts in the price series. The distributions are estimated via histograms with 10 bins, with boundaries set at the 5th and 95th percentiles of the pooled data.

Price series typically show strong trends and positive skewness, so we use a hybrid detection procedure. The procedure computes JS divergence on the empirical distributions of log-prices $\ln(P_{t})$ and monitors the relative change in segment medians:

$$\Delta m=\frac{|\mathrm{median}\left(P_{\mathrm{left}}\right)-\mathrm{median}\left(P_{\mathrm{right}}\right)|}{\mathrm{median}(P_{\mathrm{pooled}})}.$$

The combined score combines the two components:

Score=0.6×JS+0.4×Δm.

The weights give greater emphasis to distributional divergence while also accounting for large level shifts that histograms may miss. A breakpoint is identified when the score exceeds the threshold θ=0.3:

BreakattifScore>θ.

**Cluster reduction and threshold calibration.** Because the sliding window moves daily, adjacent Score values exhibit strong serial dependence. To identify distinct breakpoints from clusters of consecutive days where Score >θ, we apply non-maximum suppression (NMS) with a suppression window of ±10 trading days. Within each cluster, only the day with the maximum Score value is retained as a breakpoint. The retained breakpoints are thus separated by at least 20 days.

The threshold θ is calibrated using a time-series block bootstrap that preserves the serial dependence, volatility clustering, and heavy-tailed features of the WTI price series, rather than a simple random-walk null.

1. We generate 1000 surrogate WTI log-price series using a moving-block bootstrap (block length =50 days);
2. For each surrogate series, we apply the JS detection procedure with the same window width and histogram bins, and record the maximum Score value;
3. The threshold θ is set to the 95th percentile of the empirical distribution of these 1000 maximum Score values.

This gives θ=0.32, close to the original value of 0.30. The detected breakpoints remain nearly identical: 11 of 12 are retained, with the remaining breakpoint shifted by 2 days.

**Role of the GPR index.** The GPR index is not used in breakpoint detection; the detection procedure is data-driven and does not rely on any event labels. Rather, the GPR index is used as a post hoc validation tool. After detecting breakpoints, we check whether the corresponding GPR index is elevated relative to its historical baseline. This provides external evidence that the detected breakpoints correspond to economically meaningful events.

**Systemic penetration depth.** To quantify the impact of structural breaks beyond the magnitude of JS divergence, we define a composite metric $D_{p}$:
  $$D_{p}=\tau_{1/2}\times M,$$
  where $\tau_{1/2}$ is the half-life of the JS divergence decay following a shock (the number of days required for the JS divergence to return to 50% of its peak value), and *M* is the number of markets/variables in which the shock induces a statistically significant change at the 5% level within a 60-day window. This metric captures two dimensions of penetration depth: *persistence* ($\tau_{1/2}$) and *spread* (*M*).

### 2.7. Rolling-Window Time-Varying Analysis

To examine whether the relationships captured by MF-DCCA and PG change over time, we implement a rolling-window framework with window width W=252 trading days—approximately one year—and step size L=21 trading days—approximately one month. In each window, we compute the MF-DCCA spectrum width $\Delta h_{s}\left(t\right)$ at scale s=5 days, and the PG index $PG_{s}\left(t\right)$ at scales s∈{2,5,10} days.

This approach captures the temporal heterogeneity of market dynamics and enables systematic mapping to external shocks. We align the resulting trajectories with the timeline of major events—the 2018 US–China trade war, the 2020 negative oil price event, the 2022 Russia–Ukraine conflict, and the 2023 Red Sea crisis—enabling direct observation of how exogenous geopolitical events reshape cross-market correlations and directional asymmetry. All computations are implemented in Python 3.9, with NumPy, SciPy, and statsmodels handling the numerical and statistical work.

The key parameters and interpretation guidelines of the four methods described above are summarized in Table 4.

## 3. Results

The empirical analysis is guided by the four hypotheses pre-specified in Section 1. For the primary hypotheses H1 (MF-DCCA spectrum width peaks at 20 days) and H2 (PG sign reversal between 10 and 20 days), we report 95% bootstrap confidence intervals and *p*-values. For the secondary hypotheses H3 (MSWPE 40-day entropy drop for soybean oil) and H4 (JS breakpoint–event alignment), we apply multiple-testing corrections where applicable and interpret the results with caution. All other scale-specific comparisons and cross-commodity contrasts are treated as exploratory and presented as hypothesis-generating.

### 3.1. Scale-Dependent Multifractal Cross-Correlations

We begin by examining how the cross-correlation structure between energy and financial markets evolves across different observation scales. Table 5 reports the multifractal spectrum parameters for WTI crude oil versus the US dollar index. The spectrum width Δh rises from 0.139 at 2 days to 0.602 at 20 days, then declines to 0.380 at 40 days and 0.223 at 60 days. The evolution thus traces a full cycle: monofractal at short horizons, strongly multifractal at monthly scales, and returning to near-monofractal at quarterly horizons.

At short horizons (2–5 days), the spectrum width remains relatively narrow (Δh≈0.14–0.16), indicating a pronounced monofractal signature. Short-term markets are predominantly driven by relatively homogeneous factors—speculative flows, algorithmic trading, and microstructure frictions—that respond to small and large fluctuations in a broadly similar manner. At the 10-day and 20-day scales, however, the spectrum width jumps to 0.530 and 0.602, more than tripling from the short-horizon level. This sharp increase signals a transition to a multifractal regime: the WTI–dollar correlation is no longer independent of fluctuation amplitude, but varies systematically with the scale of price movements. At 40 days, the spectrum width falls to 0.380; at 60 days, to 0.223. The multifractal signature thus attenuates beyond the one-month horizon, though a moderate degree of asymmetry persists up to 40 days.

The source of this widening lies in the marked divergence of the generalized Hurst exponents across small and large fluctuation regimes. For small fluctuations (q=−5), $h_{xy}\left(-5\right)=0.852$ at 20 days, close to 1, indicating strong trend persistence. For large fluctuations (q=5), $h_{xy}\left(5\right)=0.250$ at 20 days, well below 0.5 and approaching anti-persistence, implying rapid reversal after large shocks. As the scale extends to 40 and 60 days, $h_{xy}\left(-5\right)$ falls to 0.761 and 0.685, while $h_{xy}\left(5\right)$ rises to 0.381 and 0.462. The gap between small and large fluctuation behaviors thus narrows considerably. This pattern indicates that at quarterly horizons, both fluctuation ranges increasingly reflect common supply–demand fundamentals, with a corresponding reduction in cross-market nonlinearity. This differentiation points to a consistent pattern: at the monthly scale, WTI–dollar co-movements exhibit significant fluctuation-size asymmetry—small fluctuations drive gradual trend accumulation, while large fluctuations trigger mean-reverting corrections. This asymmetric behavior constitutes the nonlinear foundation of medium- to long-term cross-market risk transmission.

This scale-dependent amplification reflects the differential penetration of macro-financial factors across investment horizons. At the monthly scale, monetary policy expectations, global liquidity cycles, and long-term supply–demand trends become dominant. The asymmetric response of the dollar to oil price shocks—the “commodity currency” effect—further enriches the multifractal structure. In contrast, the near-monofractal state at short horizons persists because price fluctuations are predominantly governed by inventory surprises, technical patterns, and daily positioning adjustments—factors that respond uniformly across fluctuation sizes. The attenuation of multifractality at 40 and 60 days indicates that these heterogeneous factors carry less weight over longer horizons. Prices at quarterly scales increasingly reflect broader macroeconomic fundamentals, which tend to affect small and large fluctuations more uniformly.

The contrasting results for the cross-market soybean pair (US soybeans vs. Chinese soybeans) provide a powerful counterpoint. Table 6 reports the corresponding results. The spectrum width remains consistently below 0.015 across all scales, effectively indistinguishable from zero. Moreover, $h_{xy}\left(-5\right)$ and $h_{xy}\left(5\right)$ are nearly identical at each scale, indicating that small and large price fluctuations follow the same scaling relationship—reflecting efficient cross-market arbitrage. This near-monofractal structure persists at all scales, including 40 days (Δh=0.009) and 60 days (Δh=0.009). The persistence indicates that the soybean market’s robustness to scale-dependent nonlinearity is structural, not an artifact of the sample period. This robustness persisted throughout the 2018–2020 US–China trade tensions, demonstrating that the fungible nature of soybeans—with alternative supply sources in Brazil and Argentina—preserved price equilibrium despite bilateral friction.

The contrast between WTI–DXY and US–CN soybean pairings points toward a supply-side interpretation: energy markets, with tighter supply constraints and longer adjustment lags, show stronger scale-dependent multifractality than globally tradable agricultural markets with flexible sourcing alternatives. Still, this interpretation is descriptive and hypothesis-generating rather than conclusive. The two pairings differ along multiple dimensions beyond supply elasticity—market structure, contract design, currency exposure, trading hours, regulatory frameworks, storage costs, and the nature of the paired variable. A rigorous test of the supply-elasticity hypothesis would require a broader commodity panel with direct proxies for supply elasticity (e.g., inventory-to-use ratios, production concentration indices, or structural elasticity estimates). We view the supply-elasticity interpretation as a plausible hypothesis consistent with the observed contrast, though other structural differences may also play a role.

We assess the robustness of the 20-day spectral peak with three sensitivity checks: (i) varying the detrending order from m=1 to m=3; (ii) splitting the sample into pre-COVID, COVID/Ukraine, and post-Ukraine periods; and (iii) refining the scale grid to include 15, 18, 22, and 25 days. The results, reported in Tables S1–S3, show that the 20-day peak persists across all specifications. This rules out the possibility that the peak is an artifact of detrending choice, sample selection, or scale-grid resolution.

### 3.2. Scale-Dependent Time Irreversibility

Time irreversibility refers to the statistical asymmetry of a time series under time reversal, which is a signature of dissipative dynamical systems operating away from equilibrium. In commodity futures markets, this manifests as asymmetric price responses to positive versus negative shocks: adverse news triggers more violent fluctuations than favorable news, declines are more abrupt than advances, and recoveries are more gradual. The central finding of this subsection is that this asymmetry undergoes a fundamental shift with the observation horizon.

For WTI crude oil, Table 7 reports the multiscale PG estimates. The PG irreversibility index is positive at short horizons (2–10 days), with the 5-day value reaching +0.0177 (95% CI: [0.0048,0.0306], p=0.008). The frequency component is $PG_{\mathrm{freq}}=+0.0162$ ($p_{+}=0.53$, $p_{-}=0.47$) and the magnitude component is $PG_{\mathrm{mag}}=+0.0015$. The positive PG thus comes mainly from more frequent upward moves, not larger magnitudes. This pattern is consistent with the “slow grind up, fast crash down” dynamic: upward moves occur more frequently over days to weeks, while downward moves, though less frequent, are sharper when they occur. At the 20-day scale, however, the PG reverses to −0.0267 (95% CI: [−0.0412,−0.0122], p=0.001)—a shift of approximately 0.044 relative to the 5-day value. The 20-day value decomposes into $PG_{\mathrm{freq}}=-0.0024$ and $PG_{\mathrm{mag}}=-0.0243$. The sign reversal thus comes mainly from magnitude asymmetry, not frequency. The 20-day confidence interval is entirely negative, confirming that the sign reversal is statistically significant and not a sampling artifact. Over monthly horizons, downward movements become relatively faster or more frequent than upward movements. At the 40-day and 60-day scales, the index remains negative but with diminishing magnitude (−0.0032, 95% CI: [−0.0158,0.0094], p=0.618; −0.0011, 95% CI: [−0.0145,0.0123], p=0.871), with confidence intervals crossing zero, suggesting that directional bias gradually dissipates toward equilibrium at quarterly horizons.

This cross-scale sign reversal reflects the dominance of different investor populations across time scales. Short-term speculators and momentum traders amplify upward momentum over daily to weekly intervals, generating positive PG values. At monthly horizons, commercial hedgers, producers, and institutional investors adjust positions in response to fundamental supply–demand assessments, exhibiting greater sensitivity to downside risks and producing sharper downward corrections. The near-zero PG at 60 days (approximately one quarter) is consistent with finite memory of directional bias, dissipating over a timescale comparable to a typical commodity cycle. Notably, the 20-day reversal window coincides with the one-way shipping time from the Middle East to East Asia and the global refinery inventory turnover cycle, suggesting that physical logistics rhythms have been internalized into WTI futures pricing through the hedging behaviors of East Asian market participants.

The corresponding results for US wheat are reported in Table 8 and exhibit an even more pronounced but structurally different pattern. The 5-day PG reaches +0.0229 (95% CI: [0.0086,0.0372], p=0.002), and the 10-day PG peaks at +0.0363 (95% CI: [0.0198,0.0528], p<0.001). The 10-day value decomposes into $PG_{\mathrm{freq}}=+0.0189$ and $PG_{\mathrm{mag}}=+0.0174$. Thus, frequency and magnitude contribute nearly equally to the strong positive irreversibility at this scale. This strong positive irreversibility reflects rapid price responses to weather reports, crop condition updates, and USDA announcements, with adverse reports triggering immediate upward jumps followed by gradual adjustment. At the 40-day scale, the PG reverses to −0.0073 (95% CI: [−0.0205,0.0059], p=0.278), with the confidence interval crossing zero, indicating no statistically significant directional bias at this scale. However, at the 60-day scale, a remarkable re-reversal occurs: the PG reaches +0.1142 (95% CI: [0.0826,0.1458], p<0.001). The components are $PG_{\mathrm{freq}}=+0.0117$ and $PG_{\mathrm{mag}}=+0.1025$. The positive PG comes overwhelmingly from magnitude asymmetry: the large upward price spikes at the onset of supply disruptions dominate quarterly-horizon irreversibility, not directional frequency. This exceptionally strong positive irreversibility at the quarterly horizon is attributable to the asymmetric nature of agricultural supply shocks: adverse weather or geopolitical disruptions generate rapid price spikes at the onset of the growing season, while the subsequent supply–demand rebalancing—through planting adjustments, import substitutions, and strategic reserve drawdowns—unfolds gradually over months.

The bootstrap difference test also shows that the 20-day sign reversal in WTI is statistically significant: the difference between the 10-day and 20-day PG estimates is significant (p=0.008), whereas differences among scales with the same directional sign are not (p>0.10). For US wheat, the 60-day re-reversal is also statistically significant (p=0.002), indicating a genuine shift between the 40-day and 60-day scales.

The “dual-reversal” pattern of US wheat demonstrates that directional asymmetry in agricultural commodities is not a monotonic decay process, but rather a repetitive structural alternation anchored by the growing season cycle. This contrasts with the monotonic reversal pattern observed in crude oil. Overall, time irreversibility in commodity markets is jointly determined by supply adjustment elasticity and shock accumulation patterns: energy commodities exhibit finite memory (dissipating over approximately one quarter), while agricultural commodities display cycle-anchored asymmetry (accumulating over the growing season and releasing at season-end).

### 3.3. Complexity Hierarchy Along the Soybean Crushing Chain

The soybean crushing value chain—soybeans, soybean meal, and soybean oil—provides a setting for examining how market complexity propagates along a production chain. Each processing stage alters the commodity’s physical form and economic use, generating differentiated price dynamics. Soybeans are easily storable and globally traded, with prices determined by international supply–demand; soybean meal, less storable and tied to livestock cycles, reflects regional demand; soybean oil, with dual uses as edible oil and biofuel feedstock, directly links the agricultural chain to energy markets.

Entropy declines monotonically with scale for all three products, indicating that price dynamics become more predictable and less complex as the horizon lengthens. At short scales, microstructure noise and stochastic trading shocks dominate price fluctuations, resulting in higher entropy and price paths that resemble random walks. As the scale extends, these transitory disturbances are gradually smoothed out, and fundamental trend information emerges, making price behavior more orderly and predictable. Table 9 reports the full set of multiscale entropy estimates for WTI and the soybean complex. The entropy values across all products fall roughly between 2.35 and 2.58. For d=3, the maximum possible entropy is $\log_{2}\left(6\right)\approx 2.585$ (with six possible ordinal patterns), so this range indicates a fairly high level of complexity. The distribution of the six ordinal patterns is nearly uniform, with no single pattern dominating. This near-uniform distribution indicates a high degree of ordinal unpredictability under the chosen embedding representation: no single ordinal pattern dominates, suggesting that simple deterministic structures are not readily identifiable in the price dynamics. This is consistent with, but not equivalent to, the asset-pricing notion of market efficiency, which requires additional conditions such as the absence of risk-adjusted arbitrage opportunities. We therefore interpret the entropy results as evidence of high ordinal complexity rather than market efficiency in the financial sense.

A notable exception appears at the 40-day scale: the entropy of soybean oil (2.348) drops significantly below that of soybeans (2.562) and soybean meal (2.551), a gap of approximately 0.21 bits. This divergence emerges specifically at 40 days, likely related to the institutional cycles soybean oil faces as a biofuel feedstock—the US EPA’s Renewable Fuel Standard (RFS) compliance cycles and the monthly-to-bimonthly adjustment frequency of biofuel production margins both fall within this window. Energy market trend signals become embedded in soybean oil pricing at this horizon, whereas soybeans and soybean meal lack this cross-energy-market transmission channel. At shorter scales, all three products are driven by common agricultural news and weather trading; at 60 days, all segments have synchronized to longer-term supply–demand equilibrium, and the divergence dissipates.

Table 9 also reports MSWPE results for WTI crude oil. WTI has its lowest entropy at the 20-day scale ($H_{w}=2.448$), consistent with the logistics-driven feature identified earlier. Soybean oil does not show an entropy drop at 20 days ($H_{w}=2.574$); its distinct 40-day entropy drop aligns with the biofuel policy cycle rather than WTI’s logistics rhythm. To test whether the 20-day feature propagates to the soybean chain, we compute MF-DCCA cross-correlations between WTI and each soybean complex product (Table S4). The 20-day scale shows the strongest cross-market multifractality across all three pairs, with WTI–soybean oil exhibiting the largest spectrum width (Δh=0.518), followed by WTI–soybean (Δh=0.352) and WTI–soybean meal (Δh=0.222). This ranking supports the biofuel channel interpretation: energy shocks transmit most directly to soybean oil, then to soybeans, and least to soybean meal, with soybean oil serving as the primary conduit between energy and agricultural markets.

We performed two robustness checks (Tables S5 and S6) to assess whether the 40-day entropy drop is robust to finite-sample concerns. First, using alternative embedding dimensions (d=2 and d=4), the finding that soybean oil has the lowest entropy at 40 days holds across all three *d* values (Table S5). Second, composite coarse-graining with bootstrap confidence intervals shows that the 40-day soybean oil–soybean difference is statistically significant (95% CI: [0.08,0.22] bits, p=0.032), though the gap is slightly attenuated (composite difference: 0.150 bits vs. original: 0.214 bits) (Table S6). These checks indicate that the 40-day entropy reduction is a genuine structural feature.

### 3.4. Structural Break Detection and Geopolitical Linkages

The Jensen–Shannon divergence segmentation algorithm identifies 12 structural breakpoints in the WTI price series over the sample period. Ten of the 12 breakpoints coincide with major geopolitical events—the 2018 trade war, the 2020 negative oil price event, the 2022 Russia–Ukraine conflict, and the 2023 Red Sea crisis—with the GPR Index elevated within the event windows. Classified by source, the 12 breakpoints fall into two main categories: geopolitical conflicts (approximately 60%) and supply–demand policy interventions (approximately 40%). Conventional macro-financial variables such as interest rates and exchange rates exhibit markedly weaker explanatory power at these breakpoints.

The JS divergence values range from 0.312 to 0.516. The highest values cluster around OPEC+ events—0.516 for the December 2020 agreement and 0.503 for the April 2023 production cuts—underscoring the organization’s policy decisions as the primary driver of crude oil market breaks. COVID-19 (0.483) and US election uncertainty (0.479) register comparable magnitudes, indicating that macro shocks exert impacts no less significant than supply interventions. The negative oil price event (0.381), while a landmark that overturned the futures term structure, registers a lower divergence than the pandemic and election shocks. This contrast indicates that exogenous macro uncertainties exert influence through multiple channels—demand expectations, policy pathways, and risk premia—with impact scope and persistence far exceeding those of market-internal structural adjustments.

To check whether the detected breakpoints correspond to genuine geopolitical events, we used the following procedure. Before the analysis, we compiled an external event calendar of major geopolitical and policy events from publicly available sources (news archives, policy announcements, and the GPR event database). For each event, we defined a 30-day centered window $\left[t_{\mathrm{event}}-15,\;t_{\mathrm{event}}+15\right]$ days. A detected breakpoint was counted as matched if it fell within this window. Of the 12 breakpoints detected by the JS procedure, 10 fell within the pre-specified 30-day windows of major events. To test whether this match rate could arise by chance, we ran a placebo test using the same event list and the same matching window. We randomly generated 12 break dates within the sample period and counted how many fell within the 30-day window of any major event. Over 1000 random replications, only 0.1% of the replications produced 10 or more matches (p=0.001), indicating that the alignment is highly unlikely to be coincidental.

We also tested the sensitivity of the detected breakpoints to alternative methodological choices—varying the window width (60 and 250 days), the number of histogram bins (8 and 15), and comparing with the standard Bai–Perron structural break test. The results, reported in Table S7, show that the core events (COVID-19, OPEC+ agreements, Russia–Ukraine conflict, and Red Sea crisis) are consistently detected across all specifications. Table 10 reports the detected breakpoints with their approximate 95% confidence intervals, estimated via the block-bootstrap procedure. The confidence intervals vary by event type: discrete, date-certain events (e.g., OPEC+ meetings, the negative oil price event, and the Suez Canal blockage) exhibit narrower CIs (±3 days); progressive geopolitical events (e.g., trade wars, sanctions, and the Red Sea crisis) have moderate CIs (±5 days); while shocks with gradual onset or prolonged policy uncertainty (e.g., COVID-19, China’s reopening, and the US election) exhibit wider CIs (±7 days). This variation reflects the differing degrees of timing precision associated with different types of events.

The 10 matched breakpoints correspond one-to-one with documented events, with the GPR Index elevated at each. This supports the link between geopolitical risk and structural market shifts. These breaks cover three distinct shock categories: demand-side shocks (COVID-19, China’s reopening), supply-side shocks (EU sanctions, Ukrainian attacks, Red Sea blockage), and policy expectation shifts (OPEC+ agreements, US elections). The JS divergence, as a non-parametric detection tool, captures all these shifts uniformly without requiring pre-specification of shock categories. These findings indicate that geopolitical shocks induce persistent changes in the statistical structure of commodity prices, rather than merely transient volatility spikes. Structural breaks indicate a persistent shift in the statistical properties of the price series—the mean, variance, or autocorrelation structure is altered over the remaining sample period, while the detected breaks are persistent; this does not imply an irreversible regime shift. A formal test of persistence would require out-of-sample validation or recursive estimation. The alignment of JS-identified breakpoints with geopolitical events, supported by the placebo test, indicates that geopolitical risk is not merely short-term noise. It appears to be a core driver of long-term market evolution, with implications for cross-horizon risk management and policy timing.

**Systemic penetration depth of major events.** Applying the $D_{p}$ metric to the major events in our sample:

- COVID-19 (March 2020): $\tau_{1/2}\approx 45$ days, M=5 (WTI, DXY, US Soybean, US Soybean Meal, US Soybean Oil) $\;\to \;D_{p}\approx 225$.
- Russia–Ukraine conflict (February 2022): $\tau_{1/2}\approx 35$ days, M=4$\;\to \;D_{p}\approx 140$.
- OPEC+ production cuts (April 2023): $\tau_{1/2}\approx 25$ days, M=3$\;\to \;D_{p}\approx 75$.
- Negative oil price event (April 2020): $\tau_{1/2}\approx 20$ days, M=2 (WTI only; DXY remained stable) $\;\to \;D_{p}\approx 40$.

The ranking aligns with the qualitative pattern: exogenous macro-financial shocks (COVID-19) penetrate most deeply, supply-side policy shocks (OPEC+) fall in between, and market-internal structural events (negative oil price) have the shallowest penetration.

### 3.5. Rolling-Window Analysis of Time-Varying Irreversibility

To examine whether market directionality changes over time, we perform a rolling-window estimation of the PG index for WTI crude oil with window length 252 trading days (approximately one year) and step size 21 trading days (approximately one month). Table 11 reports the rolling PG estimates for selected windows. The rolling window spans 252 trading days and advances by only 21 days per step, so adjacent estimates share nearly 90% of their data. The resulting PG trajectory is heavily smoothed and reflects accumulated directional bias over the preceding year, not an instantaneous response to any single event. We interpret changes in the rolling PG as evidence of gradual regime shifts rather than immediate event reactions. When we associate a window with a specific event, the reference is to the period covered by that window, not a direct causal effect of the event on the window estimate.

In the earliest reported window (January 2018), the PG index is negative across all scales, with the 10-day PG reaching −0.0553, reflecting downward pressure during the initial phase of US–China trade tensions. In the subsequent window (February 2018), however, the PG turns positive across all scales, with the 10-day PG rising to +0.0768, suggesting the market gradually digested the shock after initial panic subsided. The rolling window covering the COVID-19 period (October 2019 to September 2020) exhibits strongly negative PG values across all scales, with the 10-day PG reaching −0.0579, capturing the extreme bearishness of lockdowns, demand destruction, and the unprecedented negative price event. In contrast, the rolling window ending in November 2024 (which covers the period surrounding the Red Sea crisis) exhibits the strongest positive PG values across all three scales, with the 10-day PG reaching +0.0644. This elevated value reflects the accumulated directional bias over the preceding year-long window—which covers the Red Sea crisis period—rather than an immediate reaction to that event.

These time-varying patterns indicate that directional asymmetry of crude oil evolves with changing macroeconomic and geopolitical conditions—demand-side shocks push PG into negative territory, while supply-side shocks push it positive. A closer comparison of PG values within each window reveals that during extreme stress, the magnitude of asymmetry at the 10-day scale exceeds that at shorter scales (e.g., −0.0579 at 10 days versus −0.0187 at 2 days during COVID-19). This suggests directional bias is most pronounced at medium-term horizons during crises, possibly reflecting the time required for market participants to re-evaluate fundamentals and adjust medium-term positions. In quieter periods, asymmetry across scales shows smaller differences or is overall weaker.

We use a bootstrap common-horizon test to check whether the PG sign reversal and the MF-DCCA spectral peak converge on a common scale. Table S8 reports the 95% bootstrap confidence intervals for both estimates. The PG zero-crossing point is 19.4 days (95% CI: [16.8,22.5] days); the MF-DCCA spectral peak is 20.0 days (95% CI: [18.5,21.5] days). The two intervals overlap substantially, suggesting a common characteristic scale of about 20 days for WTI dynamics.

We also tested the sensitivity to window length by re-estimating the PG trajectory with a shorter window of 120 trading days (step size 21 days). The results (Table S9) show that the qualitative directional patterns are consistent with the 252-day window: the 5-day PG remains positive, the 20-day PG remains negative, and the long-scale PG returns to near zero. The shorter window produces slightly larger fluctuations, reflecting reduced smoothing, but the key finding—the 20-day sign reversal—holds for both specifications.

Time irreversibility arises from the nonlinear superposition of external shocks and market response lags at specific time scales. Following a shock, directional bias amplifies to its peak within approximately 10 days, then gradually decays. The cross-scale structure of directional bias thus carries information about shock type, transmission speed, and participant behavior patterns. Different shock types and market states produce differentiated scale-response patterns through distinct coupling pathways. For risk management, this implies that during crises, in addition to monitoring short-term volatility elevation, investors should pay particular attention to the asymmetric risk exposure of medium-term positions.

## 4. Discussion

The preceding subsections indicate that the nonlinear dynamics of commodity futures markets are shaped by three factors together: commodity supply elasticity, physical logistics rhythms, and shock penetration depth. The scale dependence pattern is shaped by supply elasticity. Logistics rhythms provide the characteristic time anchor for directional reversals, while the persistence of structural breaks depends on shock penetration depth. Together, these three dimensions provide a coherent framework for interpreting the complex system dynamics of commodity markets.

First, supply elasticity shapes the evolutionary path of cross-market linkages and directional asymmetry. The time-scale dependence of commodity markets is not homogeneous across commodities, but varies with their supply adjustment elasticity. Energy commodities such as WTI crude oil, whose supply is constrained by geographic concentration and physical logistics, exhibit cross-market linkages that become sharply more complex with expanding scales, while directional asymmetry undergoes a fundamental sign reversal at the medium-term horizon. By contrast, agricultural commodities such as soybeans, with global supply substitutability, maintain a near-monofractal cross-market structure across all scales, with directional bias close to zero. Wheat, meanwhile, displays a distinctive “positive → negative → strong positive” dual-reversal pattern, reflecting the asymmetric accumulation of shocks arising from rigidities in agricultural supply. This finding connects multifractal cross-correlation analysis and time irreversibility analysis through a single economic logic: supply adjustment elasticity determines how sensitive a commodity’s price dynamics are to scale. Higher elasticity goes with lower cross-scale heterogeneity; lower elasticity goes with stronger scale-dependent nonlinearity.

Second, a characteristic time scale of approximately 20 days is identified in WTI across two independent methodological approaches: MF-DCCA (spectrum width peaks at this scale) and PG irreversibility (sign reversal at this scale). The JS break analysis, while detecting regime shifts on approximately monthly cycles, does not estimate a characteristic frequency and therefore does not independently confirm the 20-day scale. The MF-DCCA spectrum width peaks at this scale (0.602); the PG irreversibility index undergoes sign reversal at this scale (from +0.018 to −0.027); and the majority of OPEC+ events identified by JS divergence influence the market on approximately a monthly cycle. The 20-day convergence points to a joint role for maritime transport lags and inventory buffers in shaping how the market absorbs shocks and completes directional turns. Across the two methods, linkage complexity and directional asymmetry converge on the same underlying characteristic scale, offering a consistent empirical basis for the multiscale dynamics of energy markets. We treat the logistics interpretation as a hypothesis, not a conclusive finding—economically plausible and supported by cross-method convergence, but requiring formal tests with inventory data, term-structure variables, and macro-financial controls to establish causality [52]. A different pattern emerges from the soybean processing chain. MSWPE reveals a distinct 40-day entropy drop in soybean oil. We trace this 40-day feature to the biofuel policy cycle—specifically, the U.S. EPA’s Renewable Fuel Standard (RFS) compliance and RINs generation schedule, which operates on a roughly bimonthly cycle. This stands in contrast to the 20-day feature observed in WTI crude oil. Different commodities thus exhibit different characteristic scales, shaped by their supply chain structure and institutional environment. For WTI, the characteristic scale is tied to physical logistics (shipping times and inventory turnover); for soybean oil, it is driven by regulatory compliance cycles. The 20-day and 40-day scales share a common basis: the physical and institutional rhythms of supply chains anchor commodity price dynamics at different horizons.

Third, the intensity of a shock’s reconstruction of the price statistical structure depends on its “systemic penetration depth”—the extent to which the shock transcends single-market mechanisms and diffuses through broader channels such as demand expectations, policy pathways, and risk premia into the wider macro-financial network, thereby altering the price-generating process. The JS divergence analysis reveals a critical distinction. Structural events internal to the futures market (such as the 2020 negative oil price event, JS = 0.381), despite their high news salience, exhibit lower reconstruction intensity on the price statistical structure than exogenous macro uncertainty shocks (COVID-19 at 0.483, the US election at 0.479). The negative oil price event represents an extreme failure of market mechanisms, but its impact remains relatively confined to structural repairs within the futures market itself. The COVID-19 and election shocks, by contrast, exert more comprehensive and more persistent systemic reconstruction pressures through global demand expectations, policy uncertainty, and risk appetite channels. This conclusion also links the JS break analysis with the time-varying patterns of irreversibility: demand-side shocks drive PG toward negative values, while supply-side shocks push it toward positive values. To quantify this concept, we apply the systemic penetration depth metric $D_{p}=\tau_{1/2}\times M$ (defined in the Section 2.6). The resulting values, reported in the Section 3.4, show the same qualitative pattern: COVID-19 has the highest penetration depth ($D_{p}\approx 225$), followed by the Russia–Ukraine conflict ($D_{p}\approx 140$), OPEC+ production cuts ($D_{p}\approx 75$), and the negative oil price event ($D_{p}\approx 40$). This ordering indicates that exogenous macro-financial shocks penetrate more deeply and persistently than market-internal structural events.

The findings suggest several potential applications. For portfolio allocation, the scale-dependent heterogeneity between energy and agricultural commodities points to differentiated strategy choices—mean-reversion for energy, trend-following for agriculture. The 20-day PG sign reversal offers a signal for adjusting hedge ratios and monitoring downside risk. The JS divergence breakpoints provide a framework for stress-testing against geopolitical shocks, while the MF-DCCA and PG indicators could serve as early-warning tools for regime shifts. These applications remain conceptual and require empirical validation.

This study does not include formal economic controls for the logistics hypothesis, nor does it incorporate direct measures of physical logistics—shipping times, refinery utilization rates, inventory drawdown schedules, or hedging activities. The convergence across the two methods points to a 20-day scale, but standard macro-financial variables—inventory changes, term-structure spreads, volatility measures, and open interest—could still account for the same pattern. The PG estimates at the 60-day scale should be interpreted with some caution: coarse-graining reduces the effective sample size to approximately 33 observations, and the bootstrap confidence intervals (95% CI: [−0.0145,0.0123]) are the widest among all scales, reflecting finite-sample uncertainty. The near-zero point estimate at this scale thus remains tentative. We also acknowledge that we have not performed phase-randomized surrogate or shuffled data tests to assess whether the observed multifractal and irreversibility patterns could arise from heavy tails or volatility clustering alone. Such tests, along with formal economic controls, are left for future research.

The practical implications for allocation and risk management remain conceptual, and while the 20-day scale is statistically robust, we have not tested its out-of-sample predictive power for returns, volatility, downside risk, or hedge ratios. We have not formally compared our scale-dependent measures with established commodity-finance variables—carry, basis, momentum, open interest, liquidity, inventory conditions, the dollar, and broad volatility measures. Without such comparisons, it remains unclear whether the 20-day PG sign reversal or MSWPE entropy drop adds explanatory or predictive content beyond these standard variables. Following Abudy, Kaplanski, and Mugerman (2024) [53] and Kang, Nikitopoulos, and Prokopczuk [52], future work could incorporate these variables into a formal regression framework to test whether the 20-day feature persists after controlling for economic determinants and whether our multiscale measures offer incremental value over established factors.

## 5. Conclusions

We structure the results according to the tripartite hypothesis set out in Section 2.2. Section 3.1 tests supply elasticity via MF-DCCA cross-correlations; Section 3.2 turns to logistics rhythms through scale-dependent PG irreversibility; Section 3.3 traces shock propagation along production chains using MSWPE; and Section 3.4 assesses penetration depth with JS divergence structural breaks. WTI results are presented first throughout, serving as the benchmark for cross-commodity comparisons.

This paper integrates four methodologies—multifractal analysis, irreversibility measurement, complexity quantification, and structural break detection—into a multiscale analytical framework for examining the nonlinear dynamics of commodity futures markets. Three main conclusions emerge.

First, the physical and biological constraints inherent in commodity supply are the primary source of scale-dependent heterogeneity. Energy and agricultural commodities exhibit systematic differences in the scale evolution of cross-market linkages and in their patterns of directional asymmetry—differences that are endogenously determined by their respective supply adjustment elasticities. Second, two categories of nonlinear characteristics—linkage complexity and directional asymmetry—converge at approximately 20 days for WTI crude oil. This convergence, observed across two independent approaches, points to a coupling between physical logistics rhythms and financial pricing at this scale. For the soybean processing chain, MSWPE reveals a distinct 40-day feature for soybean oil. We trace this 40-day scale to the biofuel policy cycle—specifically, the U.S. EPA’s Renewable Fuel Standard (RFS) compliance and RINs generation schedule. Different commodities thus exhibit different characteristic scales, shaped by their specific supply chain and institutional environments. Third, whether a shock induces persistent reconstruction of the price statistical structure depends on its systemic penetration depth $D_{p}$—a composite measure of its persistence ($\tau_{1/2}$) and spread (*M*). Shocks with high $D_{p}$, such as COVID-19, propagate through multiple channels and induce long-lasting structural changes, while shocks with low $D_{p}$, such as the negative oil price event, remain largely confined to the market of origin.

These findings suggest that scale-dependent heterogeneity in commodity markets is not incidental but systematically linked to supply-side fundamentals. The framework developed here—anchored in supply elasticity, physical logistics rhythms, and systemic penetration depth—offers a coherent conceptual basis for understanding the nonlinear dynamics of commodity futures markets. It has potential implications for cross-commodity allocation, multi-horizon risk management, and geopolitical scenario analysis, but these remain conceptual. We have not formally compared our measures with established commodity-finance variables, so claims about practical applications require empirical validation. The generalizability of these results to other commodity classes, such as metals and livestock, and to alternative market conditions also remains to be investigated.

## Figures and Tables

**Table 1:** Variables employed in the empirical analysis.

Category	Variable	Ticker	Description	Data Source
*Macro-financial indicators*				
Exchange rate	Dollar_Index	DXY	U.S. dollar index	Investing.com
Geopolitical risk	GPR_Index	–	Geopolitical risk index ^a^	Iacoviello ^a^
*Commodity futures prices (energy)*				
Energy	WTI_Price	CL	Crude oil futures (NYMEX)	Investing.com
*Commodity futures prices (agriculture)*				
Agriculture	US_Wheat	ZW	Wheat futures (CBOT)	Investing.com
Agriculture	US_Soybean	ZS	Soybean futures (CBOT)	Investing.com
Agriculture	US_Soybean_Meal	ZM	Soybean meal futures (CBOT)	Investing.com
Agriculture	US_Soybean_Oil	ZL	Soybean oil futures (CBOT)	Investing.com
Agriculture	CN_Soybean	B	No. 2 soybean futures (DCE)	Investing.com

^a^ GPR Index is obtained from Matteo Iacoviello’s personal website: https://www.matteoiacoviello.com/gpr.htm (accessed on 1 March 2026). All other data are collected from https://www.investing.com/.

**Table 2:** Method inputs, transformations, and economic mapping.

Method	Input Series	Transformation	Statistic	Economic Hypothesis
MF-DCCA	Raw prices	Integrated log-prices ^a^	Δh (spectrum width)	Supply elasticity
PG Index	Raw prices	Difference	PG (frequency + magnitude)	Logistics rhythms
MSWPE	Raw prices	Coarse-grained sequences	$H_{w}$ (weighted permutation entropy)	Shock propagation
JS Divergence	Raw prices	Log-prices (internal)	Score (JS + median ratio)	Penetration depth
Rolling PG	Raw prices	Difference (rolling)	PG (252/120-day windows)	Time-varying asymmetry

^a^ MF-DCCA constructs integrated profiles $X\left(i\right)=\sum_{k=1}^{i}\ln\left(P_{k}\right)$ from raw prices; the analysis is performed on these profiles. Note: All analyses use the same raw price series $P_{t}$ as the primary input. The transformation column indicates the processing applied internally by each method. Bootstrap confidence intervals and *p*-values are reported where applicable.

**Table 3:** Unified analytical framework: methods, hypotheses, and commodity focus.

Method	Hypothesis Facet	Commodity Series
MF-DCCA	Supply elasticity → cross-correlations	WTI–DXY vs. US–CN Soybean
PG Index	Logistics rhythms → directional reversals	WTI vs. Wheat
MSWPE	Shock propagation → complexity hierarchy	Soybean → Meal → Oil
JS Divergence	Penetration depth → regime persistence	WTI

**Table 4:** Summary of methodological framework.

Method	Purpose	Key Parameters	Interpretation
MF-DCCA	Cross-correlation multifractality	q∈[−5,5], s∈{2,5,10,20,40,60}, m=2	Δh>0: multifractal cross-correlations; larger Δh: richer structure
PG Index	Time irreversibility	Scale s∈{2,5,10,20,40,60}	PG>0: upward bias; PG<0: downward bias
MSWPE	Multiscale complexity	d=3, s∈{2,5,10,20,40,60}	Higher $H_{w}$: greater complexity
JS Divergence	Structural break detection	w=120, θ=0.3	JS>θ: significant distributional shift
Rolling Window	Time-varying dynamics	W=252, L=21	Tracks evolution of Δh and PG

**Table 5:** Multifractal spectrum parameters for WTI crude oil vs. US dollar index.

Scale	$h_{xy}\left(-5\right)$	$h_{xy}\left(5\right)$	Spectrum Width Δh	Local Window	Adjusted $R^{2}$
2-day	0.483	0.344	0.139	[1,4]	0.96
5-day	0.551	0.388	0.163	[2,10]	0.97
10-day	0.794	0.264	0.530	[5,20]	0.95
20-day	0.852	0.250	0.602	[10,40]	0.98
40-day	0.761	0.381	0.380	[20,80]	0.92
60-day	0.685	0.462	0.223	[30,120]	0.89

Note: The effective number of segments at each scale is: $N_{s}\left(2\right)=1011$, $N_{s}\left(5\right)=404$, $N_{s}\left(10\right)=202$, $N_{s}\left(20\right)=101$, $N_{s}\left(40\right)=50$, $N_{s}\left(60\right)=33$. All scales have $N_{s}\geq 30$; the local power-law fits yield adjusted $R^{2}$ values ranging from 0.89 to 0.98 across all scales.

**Table 6:** Multifractal spectrum parameters for US soybean vs. Chinese soybean prices.

Scale	$h_{xy}\left(-5\right)$	$h_{xy}\left(5\right)$	Spectrum Width Δh	Local Window	Adjusted $R^{2}$
2-day	0.395	0.390	0.005	[1,4]	0.94
5-day	0.421	0.411	0.010	[2,10]	0.95
10-day	0.449	0.444	0.005	[5,20]	0.93
20-day	0.473	0.461	0.012	[10,40]	0.96
40-day	0.481	0.472	0.009	[20,80]	0.94
60-day	0.498	0.489	0.009	[30,120]	0.91

**Table 7:** Multiscale PG irreversibility index for WTI crude oil.

Scale	PG Index	95% CI	*p*-Value	Significance
2-day	+0.0087	[−0.0032,0.0206]	0.152	Not significant
5-day	+0.0177	[0.0048,0.0306]	0.008	p<0.01
10-day	+0.0070	[−0.0056,0.0196]	0.274	Not significant
20-day	−0.0267	[−0.0412,−0.0122]	0.001	p<0.01
40-day	−0.0032	[−0.0158,0.0094]	0.618	Not significant
60-day	−0.0011	[−0.0145,0.0123]	0.871	Not significant

Bootstrapped with 1000 replications using a moving-block bootstrap (block length = 100 days).

**Table 8:** Multiscale PG irreversibility index for US wheat.

Scale	PG Index	95% CI	*p*-Value	Significance
2-day	+0.0084	[−0.0042,0.0210]	0.192	Not significant
5-day	+0.0229	[0.0086,0.0372]	0.002	p<0.01
10-day	+0.0363	[0.0198,0.0528]	<0.001	p<0.001
20-day	+0.0148	[−0.0012,0.0308]	0.071	Not significant
40-day	−0.0073	[−0.0205,0.0059]	0.278	Not significant
60-day	+0.1142	[0.0826,0.1458]	<0.001	p<0.001

Bootstrapped with 1000 replications using a moving-block bootstrap (block length = 100 days).

**Table 9:** Multiscale weighted permutation entropy for WTI and the soybean complex.

Scale	WTI	Soybean	Soybean Meal	Soybean Oil
2-day	2.576	2.580	2.584	2.582
5-day	2.548	2.575	2.581	2.573
10-day	2.502	2.563	2.581	2.567
20-day	2.448	2.574	2.558	2.574
40-day	2.465	2.562	2.551	2.348
60-day	2.438	2.502	2.510	2.476

**Table 10:** Structural breakpoints in WTI price series identified by JS-divergence segmentation.

No.	Date	95% CI	JS Divergence	Associated Event
1	2018-06-19	±5 days	0.338	US–China trade war escalation
2	2020-01-28	±7 days	0.483	COVID-19 pandemic outbreak
3	2020-04-21	±3 days	0.381	WTI negative oil price event
4	2020-12-29	±3 days	0.516	OPEC+ production agreement
5	2021-03-23	±3 days	0.439	Suez Canal blockage
6	2022-05-17	±5 days	0.339	EU sanctions on Russian oil
7	2022-08-09	±5 days	0.322	Biden visit to the Middle East
8	2023-01-24	±7 days	0.312	China’s post-COVID reopening
9	2023-04-18	±3 days	0.503	OPEC+ surprise production cuts
10	2023-12-26	±5 days	0.468	Red Sea crisis
11	2024-03-19	±5 days	0.451	Ukrainian attacks on Russian refineries
12	2024-11-26	±7 days	0.479	US post-election policy uncertainty

Note: Confidence intervals are estimated via block-bootstrap. CI widths vary by event type: discrete, date-certain events (e.g., OPEC+ meetings, negative oil price, Suez Canal blockage) have narrower CIs (±3 days); progressive events (e.g., trade wars, sanctions, geopolitical crises) have moderate CIs (±5 days); shocks with gradual onset or prolonged policy uncertainty (e.g., COVID-19, reopening, elections) have wider CIs (±7 days).

**Table 11:** Time-varying PG irreversibility indices for WTI crude oil (selected windows).

Window Start	Window End	PG (2-Day)	PG (5-Day)	PG (10-Day)
2018-01-03	2018-12-20	−0.0078	−0.0281	−0.0553
2018-02-01	2019-01-18	+0.0111	+0.0215	+0.0768
2018-05-01	2019-04-17	−0.0162	−0.0097	−0.0322
2018-07-27	2019-07-15	−0.0087	+0.0147	+0.0148
2019-10-02	2020-09-18	−0.0187	−0.0254	−0.0579
2020-01-30	2021-01-15	+0.0020	−0.0178	−0.0105
2020-05-06	2021-04-23	+0.0086	+0.0306	−0.0162
2021-08-31	2022-08-19	+0.0229	+0.0068	−0.0053
2022-03-16	2023-03-03	−0.0177	−0.0043	+0.0053
2023-12-12	2024-11-29	+0.0528	+0.0559	+0.0644

## Data Availability

The raw data supporting the conclusions of this article will be made available by the authors on request.

## Supplementary Materials

The following supporting information can be downloaded at: https://www.mdpi.com/article/10.3390/e28090984/s1, Table S1: Sensitivity of MF-DCCA spectrum width to detrending order (WTI–DXY); Table S2: Sensitivity of MF-DCCA spectrum width to sub-periods (WTI–DXY, m=2); Table S3: Sensitivity of MF-DCCA spectrum width to finer scale grid (WTI–DXY, m=2); Table S4: MF-DCCA cross-correlation spectrum widths between WTI and soybean complex at selected scales; Table S5: MSWPE sensitivity to embedding dimension at selected scales (soybean complex); Table S6: Composite coarse-graining MSWPE estimates and 95% bootstrap CIs at selected scales; Table S7: Robustness of JS break detection to alternative specifications; Table S8: Bootstrap confidence intervals for the common-horizon test (WTI); Table S9: Comparison of rolling PG estimates: 252-day vs. 120-day window (WTI).

## Abbreviations

MF-DCCA	Multifractal Detrended Cross-Correlation Analysis
PG	Porat–Friedland irreversibility index
MSWPE	Multiscale Weighted Permutation Entropy
JS	Jensen–Shannon divergence
GPR	Geopolitical Risk Index
WTI	West Texas Intermediate crude oil

## References

[1] Waldrop M.M. (1993). Complexity: The Emerging Science at the Edge of Order and Chaos.

[2] Mandelbrot B.B. (1983). The Fractal Geometry of Nature.

[3] Feder J. (1988). Fractals.

[4] Vicsek T. (1992). Fractal Growth Phenomena.

[5] Stanley H.E., Meakin P. (1988). Multifractal phenomena in physics and chemistry. Nature.

[6] Ftiti Z., Jawadi F., Louhichi W., Madani M.E.A. (2021). Are oil and gas futures markets efficient? A multifractal analysis. Appl. Econ..

[7] Mensi W., Vo X.V., Kang S.H. (2022). Upward/downward multifractality and efficiency in metals futures markets: The impacts of financial and oil crises. Resour. Policy.

[8] Lahmiri S. (2023). Multifractals and multiscale entropy patterns in energy markets under the effect of the COVID-19 pandemic. Decis. Anal. J..

[9] Peng C.K., Buldyrev S.V., Havlin S., Simons M., Stanley H.E., Goldberger A.L. (1994). Mosaic organization of DNA nucleotides. Phys. Rev. E.

[10] Peng C.K., Buldyrev S.V., Goldberger A.L., Havlin S., Sciortino F., Simons M., Stanley H.E. (1992). Long-range correlations in nucleotide sequences. Nature.

[11] Kantelhardt J.W., Zschiegner S.A., Koscielny-Bunde E., Havlin S., Bunde A., Stanley H.E. (2002). Multifractal detrended fluctuation analysis of nonstationary time series. Phys. A Stat. Mech. Its Appl..

[12] Podobnik B., Stanley H.E. (2008). Detrended cross-correlation analysis: A new method for analyzing two nonstationary time series. Phys. Rev. Lett..

[13] Zhou W.X. (2008). Multifractal detrended cross-correlation analysis for two nonstationary signals. Phys. Rev. E.

[14] Wu B., Jiang F., Zhang J., Liu C., Shi K. (2024). Deep multifractal detrended cross-correlation analysis algorithm for multifractals. Phys. A Stat. Mech. Its Appl..

[15] Stosic B., Stosic T. (2025). Dissecting multifractal detrended cross-correlation analysis. Phys. A Stat. Mech. Its Appl..

[16] Arianos S., Carbone A. (2009). Cross-correlation of long-range correlated series. J. Stat. Mech. Theory Exp..

[17] Lin A., Shang P., Zhao X. (2012). The cross-correlations of stock markets based on DCCA and time-delay DCCA. Nonlinear Dyn..

[18] Jiang Z.Q., Zhou W.X. (2011). Multifractal detrending moving-average cross-correlation analysis. Phys. Rev. E.

[19] Chen S., Li Q., Wang Q., Zhang Y.Y. (2023). Multivariate models of commodity futures markets: A dynamic copula approach. Empir. Econ..

[20] Dahal S., Mattos F. (2026). Exploring the presence of nonlinear deterministic dynamics in commodity prices. Agric. Food Econ..

[21] Ren Y., Tan A., Zhu H., Zhao W. (2022). Does economic policy uncertainty drive nonlinear risk spillover in the commodity futures market?. Int. Rev. Financ. Anal..

[22] Hanif W., Mensi W., Vo X.V., BenSaïda A., Arreola Hernandez J., Kang S.H. (2023). Dependence and risk management of portfolios of metals and agricultural commodity futures. Resour. Policy.

[23] Costa M., Goldberger A.L., Peng C.K. (2002). Multiscale entropy analysis of complex physiologic time series. Phys. Rev. Lett..

[24] Costa M., Goldberger A.L., Peng C.K. (2005). Multiscale entropy analysis of biological signals. Phys. Rev. E.

[25] Costa M., Peng C.K., Goldberger A.L., Hausdorff J.M. (2003). Multiscale entropy analysis of human gait dynamics. Phys. A Stat. Mech. Its Appl..

[26] Zhou Y., Xie C., Wang G.J., Gong J., Li Z.C., Zhu Y. (2024). Who dominate the information flowing between innovative and traditional financial assets? A multiscale entropy-based approach. Int. Rev. Econ. Financ..

[27] Fu J., Sun Y., Liu X., Hong B., Li S. (2024). Complexity and synchronization of carbon and new energy markets based on multiscale entropy. Energy Sci. Eng..

[28] Zhao Y., Gao X., Wei H., Sun X., An S. (2024). Early Warning of Systemic Risk in Commodity Markets Based on Transfer Entropy Networks: Evidence from China. Entropy.

[29] Xue Q., Jin X., Yu J., Liu Y. (2026). Transfer Entropy Causal Networks for Interconnectedness Analysis of Global Banking and Green Markets: A CEEMDAN-SE-KM Approach. Entropy.

[30] Bandt C., Pompe B. (2002). Permutation entropy: A natural complexity measure for time series. Phys. Rev. Lett..

[31] Zhao X., Shang P., Wang J. (2013). Measuring information interactions on the ordinal pattern of stock time series. Phys. Rev. E.

[32] Orlando G., Lampart M. (2023). Expecting the Unexpected: Entropy and Multifractal Systems in Finance. Entropy.

[33] Huang N.E., Shen Z., Long S.R., Wu M.C., Shih H.H., Zheng Q., Yen N.C., Tung C.C., Liu H.H. (1998). The empirical mode decomposition and the Hilbert spectrum for nonlinear and non-stationary time series analysis. Proc. R. Soc. Lond. A.

[34] Wu Z., Huang N.E. (2004). A study of the characteristics of white noise using the empirical mode decomposition method. Proc. R. Soc. Lond. A.

[35] Flandrin P., Rilling G., Goncalves P. (2004). Empirical mode decomposition as a filter bank. IEEE Signal Process. Lett..

[36] Stylianou O., Susi G., Hoffmann M., Suárez-Méndez I., López-Sanz D., Schirner M., Ritter P. (2024). Multiscale detrended cross-correlation coefficient: Estimating coupling in non-stationary neurophysiological signals. Front. Neurosci..

[37] De Leon Miranda J., Dolfin M., Kapetanios G., Leonida L. (2026). Detecting scale-dependent network reconfiguration via multiscale detrended cross-correlations and MST topology. Phys. A Stat. Mech. Its Appl..

[38] Zhao X., Shang P., Wang J. (2014). Measuring the asymmetric contributions of individual subsystems. Nonlinear Dyn..

[39] Zanin M., Papo D. (2025). Algorithmic approaches for assessing multiscale irreversibility in time series: Review and comparison. Entropy.

[40] Wang J., Shang P., Lin A., Chen Y. (2014). Segmented inner composition alignment to detect coupling of different subsystems. Nonlinear Dyn..

[41] Zheng D., Zhao C., Hu J. (2023). Impact of geopolitical risk on the volatility of natural resource commodity futures prices in China. Resour. Policy.

[42] Wang Y., Bouri E., Fareed Z., Dai Y. (2022). Geopolitical risk and the systemic risk in the commodity markets under the war in Ukraine. Financ. Res. Lett..

[43] Cheng D., Liao Y., Pan Z. (2023). The geopolitical risk premium in the commodity futures market. J. Futur. Mark..

[44] Yang J., Yang H., Feng Y. (2025). Quantifying the geopolitical risk resilience of commodity futures markets. Econ. Lett..

[45] Fernandes L.H.S., de Araujo F.H.A., Silva J.W.L., Tabak B.M. (2022). Booms in commodities price: Assessing disorder and similarity over economic cycles. Resour. Policy.

[46] Yang J., Feng Y., Yang H. (2025). Multiscale dynamic interdependency between China’s crude oil futures and petrochemical-related commodity futures: An integrated perspective from the industry chain system. N. Am. J. Econ. Financ..

[47] Caldara D., Iacoviello M. (2022). Measuring geopolitical risk. Am. Econ. Rev..

[48] Porat B., Friedland B. (1986). Estimation of the time irreversibility of a stationary process. IEEE Trans. Acoust. Speech Signal Process..

[49] Fadlallah B., Chen B., Keil A., Príncipe J.C. (2013). Weighted-permutation entropy: A complexity measure for time series incorporating amplitude information. Phys. Rev. E.

[50] Wu S.D., Wu C.W., Lin S.G., Wang C.C., Lee K.Y. (2013). Time Series Analysis Using Composite Multiscale Entropy. Entropy.

[51] Lin J. (1991). Divergence measures based on the Shannon entropy. IEEE Trans. Inf. Theory.

[52] Kang B., Nikitopoulos C.S., Prokopczuk M. (2020). Economic determinants of oil futures volatility: A term structure perspective. Energy Econ..

[53] Abudy M.M., Kaplanski G., Mugerman Y. (2024). Market timing with moving average distance: International evidence. J. Int. Financ. Mark. Inst. Money.

