# Supplementary material for: Multiscale Complexity and Irreversibility of Non-Stationary Time Series in Commodity Futures Markets
Source: Entropy (Basel). 2026 Sep 3;28(9):984. doi: 10.3390/e28090984 (PMC13605193; doi:10.3390/e28090984)
Supplement: Supplementary file 1 [file entropy-28-00984-s001.zip › entropy-4506573-supplementary.pdf]

entropy

Article

Multiscale Complexity and Irreversibility of Non-Stationary Time Series in Commodity Futures Markets

Xia Zhao * and Kaicheng Xie

Institute of Food and Strategic Reserves, Nanjing University of Finance and Economics, Nanjing 210023, China
* Correspondence: 16692632699@163.com

Table S1. Sensitivity of MF-DCCA spectrum width to detrending order (WTI–DXY).

**Scale**	Δh ($m = 1$)	Δh ($m = 2$)	Δh ($m = 3$)
2-day	0.148	0.139	0.128
5-day	0.172	0.163	0.151
10-day	0.551	0.530	0.497
20-day	0.618	0.602	0.570
40-day	0.398	0.380	0.352
60-day	0.235	0.223	0.205

Table S2. Sensitivity of MF-DCCA spectrum width to sub-periods (WTI–DXY, $m = 2$).

**Scale**	Δh (Pre-COVID)	Δh (COVID/Ukraine)	Δh (Post-Ukraine)
2-day	0.132	0.152	0.136
5-day	0.155	0.178	0.158
10-day	0.512	0.565	0.524
20-day	0.585	0.632	0.595
40-day	0.368	0.412	0.375
60-day	0.215	0.245	0.218

Table S3. Sensitivity of MF-DCCA spectrum width to finer scale grid (WTI–DXY, $m = 2$).

**Scale**	15-day	18-day	20-day	22-day	25-day
Δh	0.515	0.574	0.602	0.588	0.545

The 20-day scale exhibits the highest spectrum width across the refined grid.

Check for updates

Academic Editors: Cun Ji and Bo Li

Received: 1 August 2026
Revised: 31 August 2026
Accepted: 31 August 2026
Published: 3 September 2026

Copyright: © 2026 by the authors. Licensee MDPI, Basel, Switzerland. This article is an open access article distributed under the terms and conditions of the Creative Commons Attribution (CC BY) license.

Table S4. MF-DCCA cross-correlation spectrum widths between WTI and soybean complex at selected scales.

**Pair**	**5-day**	**20-day**	**40-day**	**60-day**
WTI–Soybean Oil	0.148	0.518	0.348	0.198
WTI–Soybean	0.115	0.352	0.275	0.175
WTI–Soybean Meal	0.098	0.222	0.178	0.118

The 20-day scale shows the strongest cross-market multifractality for all three pairs, with WTI–soybean oil exhibiting the largest spectrum width—consistent with the biofuel channel interpretation and the characteristic scale identified in WTI dynamics.

Table S5. MSWPE sensitivity to embedding dimension at selected scales (soybean complex).

Scale	Commodity	$d = 2$	$d = 3$ (baseline)	$d = 4$
20-day	Soybean	0.961	2.574	4.128
	Soybean Meal	0.958	2.558	4.105
	Soybean Oil	0.962	2.574	4.132
40-day	Soybean	0.955	2.562	4.112
	Soybean Meal	0.952	2.551	4.098
	Soybean Oil	0.858	2.348	3.812
60-day	Soybean	0.942	2.502	4.036
	Soybean Meal	0.945	2.510	4.042
	Soybean Oil	0.938	2.476	3.998

All values are weighted permutation entropy in bits. Maximum possible entropy: $\log_2(2!) = 1.0$ for $d = 2$; $\log_2(6) \approx 2.585$ for $d = 3$; $\log_2(24) \approx 4.585$ for $d = 4$. For $d = 2$, all values are scaled to maximum entropy 1.0; values close to the maximum indicate high complexity (near-uniform ordinal pattern distribution).

Table S6. Composite coarse-graining MSWPE estimates and 95% bootstrap CIs at selected scales.

		20-day		40-day	
**Commodity**	**Method**	**Entropy**	**95% CI**	**Entropy**	**95% CI**
Soybean	Original	2.574	–	2.562	–
	Composite	2.568	[2.542, 2.594]	2.548	[2.512, 2.584]
Soybean Meal	Original	2.558	–	2.551	–
	Composite	2.552	[2.526, 2.578]	2.539	[2.498, 2.580]
Soybean Oil	Original	2.574	–	2.348	–
	Composite	2.566	[2.540, 2.592]	2.398	[2.326, 2.470]

Composite coarse-graining with 10 shifted realizations per scale. Bootstrapped with 1000 replications (block length = 50 days). The 40-day entropy drop for soybean oil remains visible under composite coarse-graining, though slightly attenuated (composite difference vs. soybeans: 0.150 bits; original difference: 0.214 bits).

Table S7. Robustness of JS break detection to alternative specifications.

Specification	Breakpoints	Overlap with baseline	Core events detected	Notes
Baseline ($w = 120$, bins=10, $\theta = 0.32$)	12	–	All	Reference
Window $w = 60$ days	14	11 of 12 (±5 days)	All	More short-term breaks
Window $w = 250$ days	8	7 of 12 (±5 days)	COVID-19, OPEC+, Ukraine, Red Sea	Fewer, longer-term breaks
Bins=8	12	12 of 12	All	Identical to baseline
Bins=15	12	12 of 12	All	Identical to baseline
Bai–Perron test	9	8 of 12 (±5 days)	COVID-19, OPEC+, Ukraine, Red Sea	Cross-method validation

Note: “Core events” refer to COVID-19 (2020-01), OPEC+ agreements (2020-12, 2023-04), Russia–Ukraine conflict (2022-02), and Red Sea crisis (2023-12). Overlap counts are for breakpoints within ±5 days of the baseline detection dates.

Table S8. Bootstrap confidence intervals for the common-horizon test (WTI).

Statistic	Estimated value	2.5% CI	97.5% CI
PG zero-crossing point (days)	19.4	16.8	22.5
MF-DCCA spectral peak (days)	20.0	18.5	21.5

Bootstrapped with 1000 replications using a moving-block bootstrap (block length = 50 days). The MF-DCCA spectral peak at 20.0 days falls within the 95% confidence interval of the PG zero-crossing point [16.8, 22.5] days.

Table S9. Comparison of rolling PG estimates: 252-day vs. 120-day window (WTI).

Window	2-day	5-day	10-day	20-day	40-day	60-day
252-day (baseline)	+0.0087	+0.0177	+0.0070	-0.0267	-0.0032	-0.0011
120-day	+0.0052	+0.0210	+0.0035	-0.0310	-0.0045	-0.0018

Values are PG indices averaged across the full sample period. The 120-day window was chosen as a robustness check to assess whether the qualitative directional patterns are sensitive to the choice of rolling window length.
